# Exploring the Influence of a Physical Activity and Healthy Eating Program on Childhood Well-Being: A Comparative Study in Primary School Students

**DOI:** 10.3390/ijerph21040418

**Published:** 2024-03-29

**Authors:** Lilyan Vega-Ramírez

**Affiliations:** EDUCAPHYS Research Group, Department of General and Specific Didactics, Faculty of Education, University of Alicante, 03690 Alicante, Spain; lilyan.vega@ua.es

**Keywords:** physical education, healthy habits, children, body mass index

## Abstract

Childhood is a crucial stage of human development in which the lifestyles children adopt can have a significant impact on their well-being throughout their lives. The aim of this study was to analyze and compare the healthy habits and Body Mass Index (BMI) of students from a primary school that participated in a program to promote physical activity and healthy eating one year earlier with other students from two schools that had not participated in this type of program. We analyzed a sample of 287 Spanish students, aged between 8 and 12 years. A survey of healthy habits was completed, and anthropometric data were taken to determine their Body Mass Index (BMI). The questionnaire data indicated that there are some significant differences (*p* = ≤ 0.05) in the consumption of some unhealthy foods between the evaluated groups. An amount of 11% of the sample was considered obese and 26% were overweight; no significant differences were found between the groups. This study suggests that the healthy habits strategy implemented by a school improves pupils’ habits, especially in reducing the consumption of unhealthy foods. Despite the positive effects, the data indicate that these programs fall short of government recommendations, particularly in areas such as physical activity and certain dietary choices.

## 1. Introduction

Childhood is a crucial stage in human development when the lifestyles children adopt can have a significant impact on their well-being throughout their lives. The habits and behavioral patterns that children acquire at this stage can condition their physical and mental health in the future. Early life experiences have a significant impact on the course of development, influencing health, behavior, and learning throughout life [1,2]. Both favorable and unfavorable experiences in childhood contribute to the formation of brain development, which is reflected in the structure of brain architecture during the most malleable stage [2,3].

In recent decades, interest in child-related studies has increased, mainly due to growing concerns about children’s long-term health and well-being [4]. The quality of early relationships and caregiving experiences can have a significant impact on a child’s ability to establish strong and healthy relationships in later life [5,6]. It is therefore vital to understand and attend to children’s lifestyles from a multidisciplinary perspective.

In addition, nutrition and physical activity in childhood have been the subject of significant enquiry in the scientific literature [7]. On the one hand, this research underlines the importance of eating habits in childhood, pointing out that eating patterns formed in childhood often persist into adulthood [8,9], as well as sports habits [10]. Promoting these healthy habits from an early age will help children develop a healthy lifestyle that will benefit them throughout their lives [2]. It is also important to remember that adult support and example are essential for children to adopt these habits [11].

The World Health Organization (WHO) has globally implemented various policies and strategies to address and prevent unhealthy habits in children, particularly those related to unhealthy eating, lack of physical activity, and the rising rates of childhood obesity. Some notable policies include the WHO Global Strategy on Diet, Physical Activity and Health, focusing on combating childhood obesity through guidelines promoting breastfeeding, reducing consumption of added sugars and saturated fats, and advocating for physical activity and nutrition education [12]. Additionally, the European strategy for child and adolescent health and development by WHO aims to enhance the health and well-being of children and adolescents, addressing factors such as nutrition, physical activity, mental health, and safety [13]. The Global Strategy for Women’s, Children’s, and Adolescents’ Health (2016–2030), a UN-driven initiative, is more comprehensive and ambitious, emphasizing equity and aligning with the Sustainable Development Goals [14].

WHO works closely with governments, health organizations, educators, and communities to promote these healthy habits worldwide and prevent chronic diseases, such as obesity, which can have their roots in childhood [15]. Along these lines, Spain has implemented a series of policies and actions aimed at promoting healthy habits among children; some of the specific policies and measures include the following: The NAOS Strategy (Nutrition, Physical Activity and Obesity Prevention) is an initiative of the Spanish Ministry of Health that aims to promote healthy lifestyle habits in the population, with a special focus on the prevention of obesity and related diseases. This strategy includes awareness-raising campaigns, healthy eating guidelines, and physical activity promotion programs [16]. *Food Safety and Nutrition Law* is specific legislation in Spain that regulates aspects such as food advertising to children, nutrition labeling, and the composition of school food [17]. Physical activity programs in schools have been promoted to encourage regular physical activity among students [18].

On the other hand, education is positively associated with health as it helps to maintain healthy lifestyles [19]. In this sense, the Primary Education curriculum gives importance to healthy habits through the general objectives, giving value to hygiene and health, using physical education, sport, and nutrition as means to favor personal and social development [20]. Educational actions aimed at promoting healthy behaviors in the school environment are essential to prevent obesity and overweight in children, and teachers of various subjects, especially physical education teachers, play a crucial role in this promotion [11]. 

Likewise, in the Physical Education curriculum in Primary Education, a subject related to the promotion of healthy habits involving body hygiene, posture, and nutrition in both physical activity and daily life is adopted. In addition, the aim of this subject is to foster students’ motor skills, which involves the integration of knowledge, methods, attitudes, and emotions related to physical behavior [21]. Therefore, the subject of Physical Education becomes a fundamental resource in the prevention of problems derived from sedentary lifestyles such as childhood obesity.

Obesity has become a 21st century epidemic affecting the general population. Globally, in 2016, more than 41 million children under 5 years of age were overweight or obese [22]. In Spain, the situation is similar, as in 2015, 23.2% of children aged 6–9 years were overweight, and 18.1% were obese. Its growth and prevalence are a cause for alarm and concern for health institutions [22,23,24]. The PASOS study [25], representative of the Spanish population aged 8 to 16 years, found a prevalence of 33.2% of overweight children and adolescents, placing childhood obesity at 11.6% in Spain.

Several studies have pointed out that overweight children and adolescents are at increased risk of cardiometabolic diseases, musculoskeletal capacity problems, among others [22,26,27]. Technological devices in the home easily provide entertainment options that replace previously outdoor activities involving physical activity. Together with sedentary lifestyles and unhealthy foods, they contribute to weight gain and obesity [28,29], triggering detrimental health problems in the general population [22,26,27,30,31,32].

Due to this situation, different Autonomous Communities in Spain have implemented various intervention programs in Spanish schools to promote healthy habits, mainly nutrition and physical activity. These strategies have provided some positive results, mainly improving eating habits and impacting to a lesser extent on physical activity [33,34]. The effectiveness of healthy habits programs is recognized, but information on their long-term sustainability is often lacking.

One of the basic assumptions of educational systems is that what individuals learn in educational institutions can be applied in everyday life. The purpose of a training action is not for individuals to successfully pass an exam, but rather for them to be able to solve problems or face situations where they put learned knowledge and skills into play. By promoting this type of learning, the aim is not only to transmit facts, but also to develop deep understanding and the ability to apply knowledge in real-world situations.

Therefore, this study aimed to analyze and compare the healthy habits and Body Mass Index (BMI) of students from a primary school that participated in a program to promote physical activity and healthy eating with other students from two schools that do not participate in this type of program.

## 2. Materials and Methods

### 2.1. Sample

This study has a descriptive and quantitative cross-sectional design, including 145 girls and 142 boys aged 8 to 12 years (mean 9.97 ± 1.305), representing three primary schools out of a total of nine, located in the 7th district of the city of a province in Spain: School 1 (CE1), privately owned, participated in the previous school year in a healthy habits campaign, whose main objective was to promote the health benefits of daily consumption of fresh fruit and vegetables and to improve the eating habits of our society. In addition to promoting extracurricular sports activities, School 2 (CE2), which is privately owned, and School 3 (CE3), which is publicly owned, have not participated in healthy habits campaigns. Schools were selected based on convenience and availability. Informed consent was obtained from both the parents of the students following the guidelines of the data protection law and the approval of the Ethics Committee of the University of Alicante (UA-2020-09-01). Table 1 below shows the main characteristics of the schools.

### 2.2. Instruments

The questionnaire is a quantitative research technique used for data collection that allows massive applications and the collection of a large amount of information on a wide range of issues at once. It allows all responses to be recorded consistently, which facilitates data analysis.

Lifestyle Questionnaire: the questionnaire used was a modification of a survey carried out by the Conselleria de Sanitat i Consum de la Genaralitat Valenciana (Department of Health and Consumption of the Generalitat Valenciana) from the school health education program [35]. From this survey, 15 multiple-choice questions were used, filled in by the students, which asked about contents that help us to find out about eating habits, physical activity habits, and sedentary lifestyles.

Weight and height were assessed using an electronic scale and a height-measuring device. BMI was then calculated. To determine obesity and overweight within the study group, we compared the BMI values obtained with the reference distribution provided by the Orbegozo Foundation [26,36]. In this study, obesity is defined as a BMI at or above the 97th percentile, while overweight is defined as a BMI at or above the 85th percentile.

### 2.3. Procedure

The first step was to contact the school principals to present the project and request permission from the schools in writing. Once these were obtained, a letter was sent to parents informing them about the tests and requesting their permission for their children to participate. On the other hand, a meeting was held with the Physical Education teachers to explain the test procedure and to obtain their collaboration and consent to organize the data collection with them, as the tests were carried out during Physical Education hours. The lifestyle survey was given to physical education teachers who administered it in their respective classes. Once the questionnaire was applied, weight and size were evaluated by the researcher. Weight and size measurements were performed with the children wearing shorts and a t-shirt while being barefoot. The questionnaires and the collection of anthropometric data were carried out between May and June.

The IBM SPSS 26.0 software for Windows was used for data analysis, applying descriptive statistics. For the comparison of nominal variables, contingency tables were used, and Pearson’s chi-square test (X2) was used to test the association between variables. Also, one-way, factorial analysis of variance or factor ANOVA was used, post hoc Scheffer. A significance level of *p* < 0.05 was set.

## 3. Results

The most important results of this study are highlighted below, giving greater relevance to those questions that demonstrate a significant difference between the schools.

### 3.1. Sedentary Habits

Electronic devices such as television, computers, and video games are one of children’s favorite pastimes. The use of these devices is strongly associated with sedentary human behavior, especially as the number of households with multiple devices increases day by day. Table 2 shows the amount of time children use electronic devices. Our data indicate that the majority of students (60.3%) watch TV less than two hours a day. After the application of the ANOVA statistic, a significant difference was found between the CE1 and the CE3 (*p* = 0.028).

Regarding the use of computers and video games, the data indicate that approximately 80% use these devices for less than two hours and no significant differences are found between schools.

### 3.2. Sports Habits

Approximately 80% of students report doing physical activity at least once a week. Of the total sample, about half (47%) participate in sports several times a week, 19% once a week, 17% every day, and 16% never participate in sports, as shown in Table 3. When dividing the sample by school, we can see that between the variables type of school and sport frequency, there is a significance level of *p* = 0.05 between schools CE1 and CE3.

### 3.3. Eating Habits

Breakfast is one of the most important meals of the day and is essential for children to gain strength to face the day. Our data show that 56.8% of students consume milk and some industrial pastries for breakfast, 37.0% say they only drink milk, 4.9% only eat industrial pastries, and 1% say they do not eat breakfast. No significant differences were found between schools.

If we look at milk consumption, the data reveal that 45.3% of pupils drink one glass or less of milk per day, 36.6% drink two glasses of milk per day, and 18.1% drink more than two glasses per day. No significant differences were found between schools.

Table 4 shows the weekly consumption of some essential foods in child nutrition, expressed in absolute numbers and percentages. No significant differences were found between schools.

Table 5 shows the frequency of consumption of ultra-processed food products. There is a significant difference in the consumption of snacks between CE1 and CE2 (*p* = 0.009), CE1 and CE3 (*p* = 0.001). There is also a significant difference in the consumption of sugary soft drinks between CE1 and CE3 (*p* = 0.001). Another ultra-processed food product is hamburgers, where there is also a significant difference between school type CE1 and type CE2 (*p* = 0.005).

The results corresponding to the prevalence of overweight and obesity indicate that the average values of the BMI increase with age in the case of boys, while in girls there is an increase between eight and ten years as well as a slight decrease. Table 6 and Table 7 show a list of students’ anthropometric characteristics.

The BMI data revealed 11% of this sample was considered obese and 26% was overweight. When we separate the sample into groups according to schools and BMI, we observe that obesity rates are quite similar in the three schools (CE1 = 10.9%; CE2 = 12.1%; CE3 = 11.6). As for overweight, CE1 shows a slightly lower percentage (21.8%) compared to CE2 (28.6%) and CE3 (27.4%). When ANOVA analysis was performed, no significant differences were observed between students in the different schools.

## 4. Discussion

Therefore, the aim of this study was to analyze the healthy habits and growth of pupils in a primary school that participated in a program to promote healthy lifestyles and pupils in two other schools not participating in these strategies.

### 4.1. Sedentary and Physical Activity Habits

Two-thirds of the world’s population maintain sedentary practices, a result of the urbanization of the population. Currently, the Spanish population lives in large cities, specifically seven out of every ten Spaniards live in urban areas and in the case of Alicante approximately 75% [37]. This leads to reduced recreational spaces, a greater flow of vehicles, and adding to this situation, the excessive use of electronic devices for the entertainment of children and adolescents such as computers, television, or video games, which in some cases hinder and in other cases reduce children’s play in the street [38]. There are more and more new activities carried out in free time such as watching television, playing video games, or browsing the internet [18,39]. In this sense, Paniagua [40] tells us that all Spanish homes have at least one television; at 6 years old, approximately 20% of children have electronic devices in their room; between 9 and 17 years old, 30% of minors report the presence of a television in their room. In other countries such as the United States, children under eight years of age mostly live in a home with a television and mobile multimedia devices [41]. On the other hand, in recent years, the number of children who have their own tablets has increased (from 7% in 2013 to 42% in 2017) [42]. This confirms that the hours that children spend in front of these electronic devices take away time from carrying out other more active recreational activities, because they are spending more time on television, computers, or video games than reading for entertainment or engaging in physical activity [40,41]. Based on the results of our survey, especially on the questions about the frequency of daily hours in front of a computer, television, and video game, we can say that for the children in the sample, television continues to be the preferred entertainment over videos, games, and the computer. The data indicate that most of the students in the sample watch television less than 120 min/day. Data differ from other studies, where the average daily television consumption among children and adolescents is greater than two hours a day [41,42]. Minors spend 126 min/day in front of the television on school days and 150 on weekends [40]. On the other hand, the PASOS study [25] carried out in Spain revealed that the average screen time is very high, both on weekdays (193.9 min per day on average) and on weekends (288.4 min on average). One of the most relevant findings of our research has been the difference that we have found between educational centers. Children in school 1 watch less television than those in school 3. This difference may be related to the school’s healthy habits campaign, which focuses on healthy behaviors, or to socio-cultural differences between families.

On the other hand, the practice of physical activity during childhood and school years plays a significant role in promoting physical, motor, and cognitive development, in addition to maintaining health [43]. Regarding the frequency of sports practice outside of school, according to our research, the studies carried out by Rodríguez et al. [44], and the ALADINO Study [45], around 70% of children between 8 and 10 years old do some physical activity outside of school hours, at least once a week, which are data that coincide with ours. Likewise, these results are reflected in the study of German schoolchildren between 7 and 11 years old, where 70% are linked to a sports club [21]. In this regard, the current recommendation suggests 60 min a day of physical activity [18]; this prevalence in the European Union ranges from 11% to 46% in European children and from 5% to 29% in the case of girls [46]. These data coincide with ours where approximately 17% of students carry out daily physical activity. On the other hand, the PASOS study [47] shows that the percentage of the Spanish child and adolescent population that reaches the WHO recommendation regarding the practice of moderate or vigorous physical activity is only 36.7%.

Although the data on the practice of physical activity are generally lower than the recommendations, CE1 has a higher percentage of students who practice some sports activity outside of school hours compared to CE2 and with a significant difference compared to CE3. This may be related to the offer of extracurricular sports activities or, as mentioned above, this difference may be due to the healthy habits campaign. It is worth mentioning that some studies indicate that strategies to promote physical activity have had less impact than healthy eating strategies [33,34].

### 4.2. Eating Habits

Considering that nutrition directly influences the development and growth of children, a good diet can reduce avoidable chronic diseases and even improve quality of life [8,9]. As we have observed in the results of our research, which agree with the research of the Aladino study [45], the intake and frequency of some foods such as raw vegetables and boiled vegetables have revealed to us that the subjects show a low consumption of these foods. Regarding the consumption of fruit, although it is greater than that of vegetables, this is still a low consumption with respect to nutritional recommendations. As we have observed, the results of our research agree with the ALADINO study [48], that taking into account the recommendation to include five servings of fruit and/or vegetables daily in the diet, the study figures indicate that this aspect of the diet can be improved. Only about 30% of schoolchildren eat fresh fruit daily and 13.0% eat vegetables daily. The results also indicate that CE1 registers a greater consumption of these foods, despite not finding significant differences between educational centers.

When analyzing the consumption of some high-calorie foods, our results show that the intake of snacks is consumed three to six times by approximately 20% of students, a figure lower than that presented by the Aladino study [45,48], which reports that more than 70% of schoolchildren consume this type of food between one and three times a week. When we conduct an analysis by school, we see that there is a significant difference between CE1, which consumes these products to a lesser extent than CE2 and CE3. This difference could be explained by the participation of CE1 in the healthy habits strategy. Other high-calorie products analyzed were sugary soft drinks. In relation to our sample, approximately 50% consumed sugary soft drinks between one and three times per week, a figure higher than that presented by the ALADINO study [48] (36.8%). Sugary soft drinks are consumed to a lesser extent in CE1 with a significant difference in daily consumption compared to CE3. These results are alarming, since the consumption of unhealthy foods is considered a risk factor for non-communicable diseases [9]. Also, here we find a significant difference between school 1 and schools 3; these differences may be related for the reasons mentioned above, where school 1 is carrying out a healthy diet campaign for students. Hamburgers are widely enjoyed around the world, appreciated for their convenience, affordability, and delicious taste. In Spain, according to MAPA [49], the weekly consumption of hamburgers exceeds three million units. Despite their popularity, hamburgers are considered unhealthy, due to their high levels of saturated fats and low fiber content [50]. The consumption of fast food, such as hamburgers or hot dogs, occurs daily in 10% of adolescents and weekly in 41% [51]. Our data report that students mostly consume this product between one and three times a week. CE1 presents lower consumption of this product with a significant difference with CE2. Food habits and preferences are formed from childhood but are modulated by the influence of numerous factors, among which the influence of the family, the socioeconomic environment, and the school environment stand out, among others [39,52].

### 4.3. Height, Weight Growth, and Body Mass Index

Growth depends on the genotype and external factors such as the socioeconomic circumstances of a population, as well as nutrition and the absence of diseases [53].

The weight and height of the subjects in the sample, in both sexes, follow a normal growth development pattern when compared with the standards of other studies of the Spanish population [54,55]. The “growth spurt” that occurs during puberty is earlier in girls than in boys [55]. This is also reflected in the case of the sample of this study, where girls at the age of 8 to 9 years present a greater increase in height in relation to boys, their height beginning to stabilize at age 12; on the other hand, there is an increase in the height of children at the age of 11 to 12 years.

Body weight, like height, has increased in recent decades in the Spanish population with the consequent increase in BMI [48]. The ALADINO study in 2019 [48] showed a prevalence of obesity of 17.3% and estimated overweight was 23.3% for the age group between 6 and 9 years. The PASOS study [25] shows that 22.3% of children between 8 and 12 years of age are overweight and 13.3% are obese. Our study reveals a lower figure (11%) in obesity. The same does not happen with overweight, since there is a prevalence of 26% in our students, a figure higher than that of this study. No significant differences were found between the schools in the sample, with similar percentages of obesity. However, in terms of overweight, school CE1 has a lower prevalence percentage, which is not significant, compared to the other two schools.

As we have mentioned previously, obesity is considered by the WHO as the epidemic of the 21st century due to the connotations it has in terms of its impact on health. The increase in obesity in children aged 6 to 11 has more than doubled since 1960, accelerating its growth in the late 1980s in all countries among young people and adults [18,56]. Taking into account that this is a health problem that has been increasing in Spain as well as in other advanced Western countries and in countries with less economic progress [18,57], international organizations such as the World Health Organization and the Ford Task Force International Organization and the health systems of different countries have launched various programs in order to prevent this disease, since in the childhood stage of life, overweight and obesity are not associated with higher short-term mortality rates, but with a higher risk in adulthood [55]. Reviewing the reports of overweight and obesity from the last 10 years provided by the ALADINO study [48], they reflect a stabilization of the increasing trend of body mass index (BMI) in children and adolescents. This fact may be associated with the strategies emanating from government agencies that focus their campaigns on combating a sedentary lifestyle and unhealthy eating habits, two of the primary factors that have influenced the increase in obesity [53,57].

One of the limitations of this study lies in the absence of previous data on the healthy habits of the students who were part of the healthy intervention program. The availability of this information would have provided a more solid basis for a more accurate assessment of the impact of the program.

Another limitation to mention is that some factors have not been taken into account, such as the socio-economic situation of the pupils, which would allow us to obtain more complete information on healthy lifestyles.

As future lines of research, we consider addressing the long-term effects of school programs aimed at promoting physical activity and healthy eating in childhood, analyzing the impact on the health and well-being of participants as they progress through adolescence and adulthood, identifying common barriers and facilitators of participation.

## 5. Conclusions

Regarding the objective of our study, we can say that the healthy habits strategy improves the healthy habits of students, especially in the consumption of unhealthy foods, with significant differences compared to the rest of the students.

The practice of extracurricular physical activity is more frequent in students from the school that participated in the healthy habits’ strategy, showing significant differences with the public school.

Overweight and obesity are high in the sample of schoolchildren; no significant differences were found between children from the different schools.

Although the data show positive impacts of healthy programs on students, it is evident that they do not fully comply with the recommendations of government agencies in the daily consumption of fruits and vegetables and the frequency of physical activity.

## Figures and Tables

**Table 1:** Main characteristics of the schools.

Characteristics	CE1	CE2	CE3
Extracurricular activities	Psychomotricity, chess, sports gymnastics, basketball, indoor football, judo, ballet, theatre, IT, English.	Judo, football, belly dancing, English, artistic workshop	Judo, ballet, theatre, Mini basket, aerobics and IT
Geographical origin of pupils	A total of 20% of pupils come from areas close to the school, 80% from areas far from the school.	A total of 90% of the pupils belong to the neighborhood where the school is located.	Most of the pupils live in the vicinity of the school.
Parent’s level of education	Most of the parents have a high average economic situation. The proportion of parents with medium or higher education is 15%.	Most of the parents belong to a middle socio-economic level, who have completed their basic and secondary education.	There is social diversity in the school, 80% of parents are middle class and 20% are upper and lower class. Parents’ level of education is low.
In the area where the three schools are located, there is a private sports club that offers activities such as soccer, basketball, judo, skating, rhythmic gymnastics, and karate.			

In their curricular programs, the three schools dedicate a didactic unit to the contents of healthy habits in physical education classes with a duration of 3 to 5 sessions.

**Table 2:** Habits of sedentary lifestyles according to educational centers.

Hours of Sedentary		CE1		CE2		CE3		Total	
		n	%	n	%	n	%	n	%
Hours TV	Less than 2 h	69	68.9	52	57.1	52	54.7	173	60.3
	Between 2 and 3 h	29	28,7	29	31.9	31	32.6	89	31.0
	More than 3 h	3	3.0	10	11.0	12	21.6	25	8.7
Hours PC and VG	Less than 2 h	82	81.2	70	77	69	72.6	221	77.0
	Between 2 and 3 h	14	13.9	12	13.1	19	20.0	45	15.7
	More than 3 h	5	4.9	9	9.0	7	7.4	21	7.3

**Table 3:** Frequency of sports practice according to educational centers.

Sport Frequency	CE1		CE2		CE3		Total	
	n	%	n	%	n	%	n	%
Never	7	6.9	19	20.9	20	21.1	46	16.0
One a week	19	18.8	15	16.5	22	23.2	56	19.5
Several times a week	56	55.4	44	48.4	35	36.8	15	47.0
Every day	19	18.8	13	14.3	18	18.9	50	17.4
Total	101	100	91	100	95	100	287	100

**Table 4:** Consumption of essential foods in child nutrition according to educational centers.

Consumption of Essential Foods		CE1		CE2		CE3		Total	
		n	%	n	%	n	%	n	%
Vegetables	Never	35	34.7	34	37.4	37	38.9	106	36.9
	1 to 3 times a week	50	49.5	37	40.7	39	41.1	126	43.9
	3 to 6 times a week	7	6.9	10	11.0	11	11.6	28	9.8
	Every day	9	8.9	10	11.0	8	8.4	27	9.4
Fruits	Never	26	25.7	17	18.7	29	30.5	72	25.1
	1 to 3 times a week	20	19.8	32	35.2	17	17.9	69	24.0
	3 to 6 times a week	55	54.5	41	45.1	49	51.6	145	50.5
	Every day	0	0.0	1	1.1	0	0.0	1	0.3
Meat	Never	52	51.5	40	44.0	36	37.9	128	44.6
	1 to 3 times a week	42	41.6	40	44.0	43	45.3	125	43.6
	3 to 6 times a week	7	6.9	11	12.1	16	16.8	34	11.8
	Every day	0	0.0	0	0.0	0	0.0	0	0.0
Fish	Never	8	7.9	8	8.8	6	6.3	22	7.7
	1 to 3 times a week	56	55.4	37	40.7	52	54.7	145	50.5
	3 to 6 times a week	32	31.7	40	44.0	31	32.6	103	35.9
	Every day	5	5.0	6	6.6	6	6.3	17	5.9
Legumes	Never	27	26.7	20	22.0	21	22.1	68	23.7
	1 to 3 times a week	49	48.5	51	56.0	47	49.5	147	51.2
	3 to 6 times a week	25	24.8	20	22.0	27	28.4	72	25.1
	Every day	0	0.0	0	0.0	0	0.0	0	0.0
Dairy products (cheese, yogurt	Never	44	43.6	15	16.5	38	40.0	97	33.8
	1 to 3 times a week	32	31.7	46	50.5	35	36.8	113	39.4
	3 to 6 times a week	25	24.8	30	33.0	22	23.2	77	26.8
	Every day	0	0.0	0	0.0	0	0.0	0	0.0

**Table 5:** Frequency of consumption of ultra-processed foods by educational center.

Consumption of Ultra-Processed Foods		CE1		CE2		CE3		Total	
		n	%	n	%	n	%	n	%
Hamburgers	Never	20	19.8	9	9.9	16	16.8	45	15.7
	1 to 3 times a week	69	68.3	55	60.4	61	64.2	185	64.5
	3 to 6 times a week	12	11.9	27	29.7	18	18.7	57	19.9
	Every day	0	0.0	0	0.0	0	0.0	0	0.0
Snacks	Never	19	18.8	9	9.9	10	10.5	38	13.2
	1 to 3 times a week	69	68.3	49	53.8	50	52.6	168	58.5
	3 to 6 times a week	8	7.9	27	29.7	23	24.2	58	20.2
	Every day	5	5.5	6	6.6	12	12.6	23	8.0
Sugary drinks	Never	28	27.7	20	22.0	17	17.9	65	22.6
	1 to 3 times a week	52	51.5	52	57.1	48	50.5	152	53.0
	3 to 6 times a week	20	19.8	12	13.2	16	16.8	48	16.7
	Every day	1	1.0	7	7.7	14	14.7	22	7.7
Candies	Never	36	35.6	30	33.0	27	28.4	93	32.4
	1 to 3 times a week	50	49.5	48	52.7	49	51.6	147	51.2
	3 to 6 times a week	15	14.9	13	14.3	19	20.0	47	16.4
	Every day	0	0.0	0	0.0	0	0.0	0	0.0

**Table 6:** Distribution of weight, size, and body mass index (BMI) statistics by age of boy.

			Boys						
	Weight/kg			Height/cm			BMI		
Age	Mean	sd	*p* *	Mean	sd	*p* *	Mean	sd	*p* *
8 years	35.5	7.6	97	1.36	0.05	92	19.0	2.90	84
9 years	37.1	7.9	90	1.37	0.06	75	19.5	3.05	78
10 years	39.2	9.8	88	1.41	0.07	75	19.4	3.46	70
11 years	45.7	9.5	88	1.46	0.08	75	19.6	3.06	68
12 years	50.6	10.9	88	1.55	0.09	88	20.7	2.98	65

* percentile.

**Table 7:** Distribution of weight, size, and body mass index (BMI) statistics by age of girl.

			Girls						
	Weight/kg			Stature/cm			BMI		
Age	Mean	sd	*p* *	Mean	sd	*p* *	Mean	sd	*p* *
8 years	28.8	2.58	60	1.31	0.05	75	16.6	1.0	46
9 years	37.4	7.89	78	1.38	0.06	75	19.4	3.4	73
10 years	42.5	9.63	80	1.45	0.06	79	20.1	3.7	74
11 years	45.1	8.47	77	1.50	0.05	78	19.8	3.1	60
12 years	45.7	10.7	70	1.53	0.05	71	19.3	4.1	54

* percentile.

## Data Availability

The data presented in this study are available on request from the corresponding author.
